# Effect of Superplasticizer Dosage on Mechanical and Durability Properties of Low-Volume Steel Microfiber Reinforced Self-Compacting Concrete

**DOI:** 10.3390/ma19163557

**Published:** 2026-08-21

**Authors:** Jinchi Wu, Conteh Santigie Morlor, Donghua Yu, Linbin Wang, Gengying Li, Jingjing Huang

**Affiliations:** 1 Hubei Industrial Construction Group Co., Ltd., Wuhan 430076, China; 13477082009@139.com (J.W.); 13871422196@139.com (D.Y.); 2 College of Water Conservancy and Civil Engineering, South China Agricultural University, Guangzhou 510642, China; 18929541979@163.com; 3 School of Civil Engineering and Transportation, Foshan University, Foshan 528225, China; 4 School of Civil Engineering & Architecture, Wenzhou Polytechnic University, Wenzhou 325035, China

**Keywords:** self-compacting concrete, steel microfibers, mechanical properties, drying shrinkage, freeze-thawing resistance, railing structure

## Abstract

**Highlights:**

- Increasing SP from 0.8% to 1.5% reduces strength and durability by increasing porosity and refining capillary pores, as confirmed by MIP analysis.
- Steel microfibers at 0.2–0.4 vol.% enhance mechanical properties and reduce drying shrinkage through crack-bridging and matrix restraint.
- Fiber reinforcement improves freeze–thaw resistance by mitigating internal damage accumulation during cyclic freezing and thawing.
- Excessive SP weakens fiber–matrix interfacial bonding, diminishing the toughening efficiency of steel fibers.
- The optimal cost-effective mixture for railing structures is 0.3 vol.% steel fibers with 0.8 wt.% SP, balancing mechanical properties, durability, and castability.

**Abstract:**

This study investigates the effects of low-volume steel microfibers (0–0.4 vol.%) and superplasticizer (SP) dosage (0.8 wt.% and 1.5 wt.%) on the mechanical and durability properties of self-compacting concrete (SCC) for railing structures, with a constant water-binder ratio of 0.28. Fresh (slump flow), mechanical (compressive strength up to 90 days, 28-day flexural strength), durability (drying shrinkage, freeze–thaw resistance after 200 cycles), and microstructural (mercury intrusion porosimetry) properties were evaluated. SP enhances flowability while steel fibers reduce it. All mixtures except that with 0.8% SP and 0.4% fibers meet the workability requirements of Chinese standard JGJ/T 283-2012 for SCC. Compressive and flexural strengths generally increase with fiber content but decrease when the SP dosage rises from 0.8% to 1.5%. Steel fibers effectively reduce drying shrinkage and improve freeze–thaw resistance, as indicated by higher relative dynamic elastic moduli and lower mass loss after 200 cycles. Microstructural analysis reveals that the higher SP dosage (1.5 wt.%) significantly increases porosity, which explains the observed higher shrinkage and lower strength. Considering mechanical properties, durability, and castability, the SCC mixture with 0.3 vol.% steel fibers and 0.8 wt.% SP is recommended for railing structure applications.

## 1. Introduction

For safety purposes, concrete railings are installed along bridges and road sections. According to standards [1,2], their performance must satisfy four key requirements: preventing vehicle departure from the roadway, ensuring occupant protection, redirecting errant vehicles back onto the road, and resisting railing penetration. Nevertheless, as a quasi-brittle material, concrete exhibits poor tensile behavior. In most instances, its tensile strength remains below 12% of its compressive strength [3,4,5], rendering it highly susceptible to the initiation and propagation of cracks of various sizes within the railing body. These cracks, induced by external loads or shrinkage (both plastic and drying), in turn compromise the railing’s ability to withstand aggressive agents [6].

Extensive research suggests that numerous fiber types are capable of reducing concrete brittleness while upgrading multiple performance attributes, including tensile strength, flexural strength, thermal shock resistance, and toughness [7,8,9,10,11,12]. Common fiber materials include steel, carbon, and polymers. Due to its superior strength, robust durability, and excellent compatibility with cementitious matrices, steel fiber has become the most commonly used variety [3,4,6,8,9,10,11,13]. The bridging mechanism of steel fibers—covering debonding and pullout processes—can absorb considerable energy. Consequently, steel fiber-reinforced concrete demonstrates substantially enhanced post-cracking resistance, tensile strength, and deformation capacity, as well as greater toughness, energy absorption, and improved impact and wear resistance [9,11,14,15]. Existing references have consistently shown that steel fibers effectively limit crack propagation widths, leading to improved durability performance because narrower cracks reduce concrete permeability and thus enhance the corrosion protection of embedded steel reinforcement [4,9,13]. At present, steel fibers are produced at industrial scale and readily available commercially for use in civil, hydraulic, traffic, and underground engineering projects [16,17,18]. However, previous investigations have largely focused on fiber dosages above 1.0 vol.% to produce high-ductility mixtures. Despite the potential benefits of sub-1.0 vol.% fiber fractions for many infrastructure applications, experimental evidence concerning such low-volume formulations remains very scarce [18,19,20]. Recent studies have begun to address this gap; for instance, Ahmad et al. [21] demonstrated that steel fibers at dosages of 0.19–0.38 vol.% can effectively reduce shrinkage strain by up to 38% in fiber-reinforced concrete. Additionally, Franco-Quiñonez et al. [20] and Xia et al. [22] have recently reported that low-volume steel fibers (below 0.5 vol.%) can improve mechanical properties without severely compromising workability, yet these studies were conducted on conventional vibrated concrete rather than SCC, leaving the applicability of such low dosages in SCC largely unexplored.

Despite the superior performance of steel fiber-reinforced concrete noted above, some studies have indicated that adding steel fibers to concrete mixtures could result in higher water absorption and increased porosity in the hardened material [13,23]. Moreover, the addition of steel fibers may greatly increase the cost of concrete which limits its application on the railing structure since the price of steel fibers (more than 10 RMB/kg in China) is much higher than that of the raw materials of concrete (about 0.1~0.5 RMB/kg in China) and a large mass of steel fibers (with a specific gravity of 7.8 g/cm^3^) is required even with a low dosage [3,24]. For example, the incorporation of 1.0 vol.% (the normal dosage) steel fibers may increase the price of concrete by about 780 RMB/m^3^. The price of ordinary concrete (C30–C50) is about 350~450 RMB/m^3^; thus, the addition of 1.0 vol.% steel fiber may increase the price of concrete by 1.7~2.2 times. Therefore, a low volume of steel fibers will be used to reduce the cost of concrete for use in railing.

**Figure 1:**
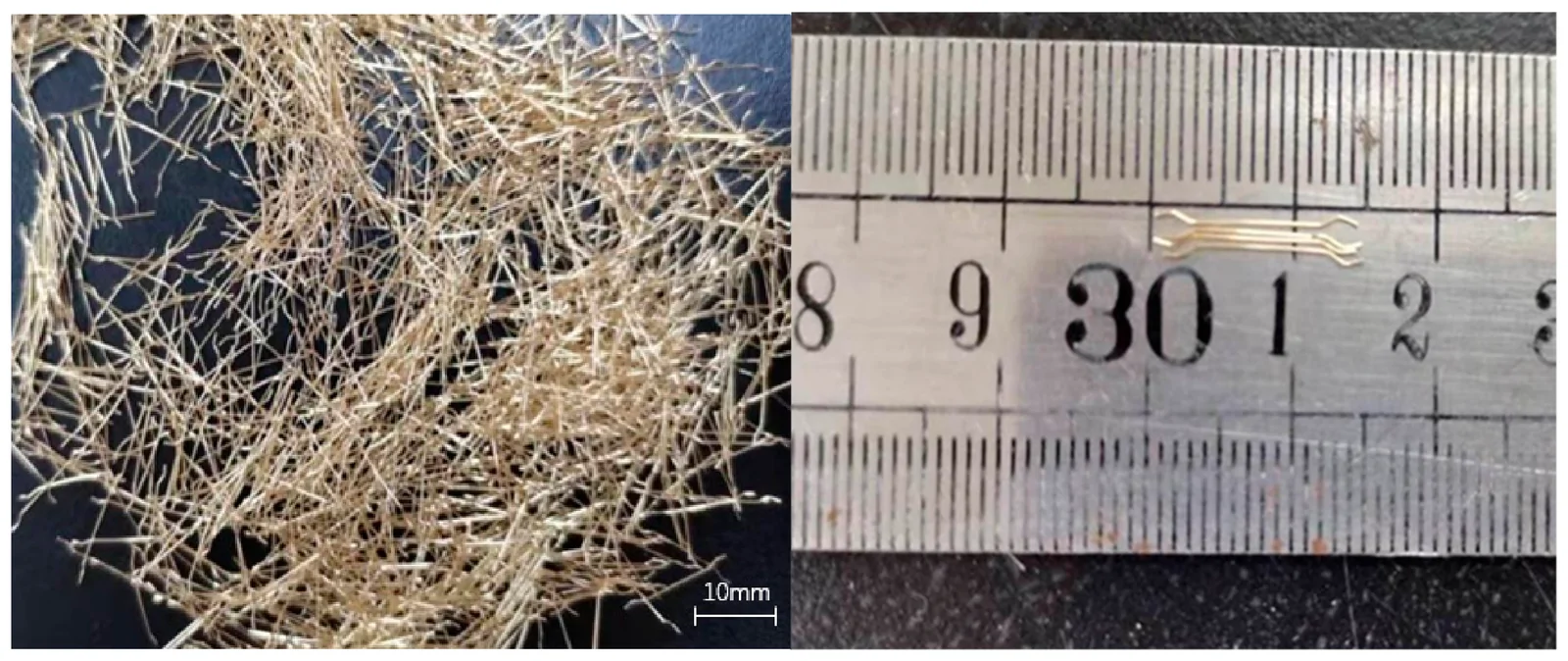
Photographic views of hooked steel microfibers.

Additionally, it is reported that the incorporation of steel fibers significantly reduced the flowability of concrete [25], which may further limit the application of steel fibers in the railing structure. It is well known that the railing structure with a special shape (Figure 1) of narrow at the top (12 cm) and wide at the bottom (20~25 cm) is generally constructed by using reinforced concrete. Poor flowability of steel fiber concrete could compromise both the mechanical properties and durability of railing structures. To address this limitation, self-compacting concrete (SCC) can be employed, since SCC, as its name implies, requires no vibration to reach full compaction [17,26,27,28]. Widely used in construction, SCC offers four main benefits: (a) lower noise from vibratory operations; (b) reduced placement expenses; (c) accelerated construction schedules; and (d) diminished need for specialized workers. Additionally, SCC is considered eco-friendly due to its high content of fine particles, which often come from recycled sources like marble waste or fly ash, and it also contributes to lower execution costs while producing a more durable concrete [4]. Recently, Murtaza et al. [29] explored the use of cement kiln dust (CKD) as a partial cement replacement in SCC, demonstrating that 5–10% CKD incorporation can yield satisfactory freeze–thaw and carbonation resistance, further highlighting the potential of SCC as a sustainable construction material. SCC can readily attain high compressive strengths; nevertheless, its paste volume is larger than that of ordinary concrete, resulting in a lower elastic modulus and greater drying shrinkage [30,31]. As noted by several researchers, incorporating steel fibers into SCC can increase the elastic modulus and reduce drying shrinkage [23,32,33,34].

Despite these recognized benefits, the application of steel fibers in SCC presents specific challenges that have not been fully resolved. SCC is characterized by its high fluidity, passing ability, and segregation resistance, which are essential for filling complex formwork and densely reinforced sections without vibration [25,27]. The incorporation of fibers, however, tends to reduce flowability due to increased internal friction and fiber–matrix adhesion [22]. Consequently, most previous studies on steel fiber-reinforced SCC have concentrated on fiber dosages exceeding 0.5 vol.% [32,35], while systematic investigations on sub-0.5 vol.% fiber fractions remain scarce. Moreover, the interaction between low-volume fibers and superplasticizer (SP) dosage—a critical factor governing both the fresh and hardened properties of SCC—has received limited attention. Although SP is commonly used to restore workability lost due to fiber addition, excessive SP can introduce adverse effects such as increased porosity, reduced strength, and greater drying shrinkage [36,37]. The combined effects of low-volume steel fibers and varying SP dosages on the comprehensive performance of SCC—including mechanical properties, drying shrinkage, freeze–thaw resistance, and microstructure—have not been systematically elucidated. This knowledge gap is particularly relevant for applications such as concrete railings, where cost-effectiveness and moderate performance enhancements are prioritized over ultra-high ductility, and where the reinforcement spacing is relatively large (longitudinal bar spacing ≈ 100–150 mm), making the passing ability requirement less stringent than for heavily reinforced sections.

The motivation of this work is to develop a Grade 50 SCC with a high performance-price ratio for railing structures, using two normal dosages (0.8% and 1.5% by weight of cement) of polycarboxylate ether-based superplasticizers (SP). Superplasticizer (SP)—a high-range water-reducing admixture—is a low-molecular-weight, water-soluble polymer capable of achieving significant water reduction (up to 30%) in concrete mixtures to obtain the desired slump [38,39]. The incorporation of SP into SCC represents an important advancement in concrete technology, as it produces a homogeneous and cohesive concrete with little to no tendency for segregation or bleeding. The efficiency of a given SP dosage is influenced by the water-to-cement ratio of the mix. Therefore, in the present study, the water-to-binder ratio was maintained constant at 0.28. Moreover, to reduce the cost of SCC and get good flowing properties, a low volume of steel microfibers (0~0.4 vol.%) was added. Despite the above-mentioned advantages, the combined effects of low-volume steel fibers and varying SP dosages on the fresh, mechanical, and durability properties of SCC for railing applications have not been systematically investigated. In particular, the following critical questions remain unanswered: (1) Can low-volume steel fibers (≤0.4 vol.%) effectively enhance the mechanical and durability performance of SCC without compromising its self-compacting characteristics? (2) How does SP dosage modulate the effectiveness of low-volume fiber reinforcement? (3) What are the microstructural mechanisms underlying the combined effects of fibers and SP on SCC performance? This study therefore aims to fill this gap by comprehensively evaluating the slump flow, compressive and flexural strengths, drying shrinkage, freeze–thaw resistance, and microstructure (via MIP) of SCC mixtures containing low-volume steel microfibers (0–0.4 vol.%) and two SP dosages (0.8% and 1.5%). The findings are expected to provide a practical basis for designing cost-effective and durable SCC railings with balanced performance.

## 2. Materials and Methods

### 2.1. Materials, Mixing and Curing

The present study utilized ordinary Portland cement, fly ash (FA), siliceous sand, granite coarse aggregate, steel fiber, and superplasticizer. The ordinary Portland cement (Type 42.5R), supplied by Hailuo Cement Co., Ltd. (Wuhu, China), complies fully with the requirements of BS EN 197-1:2024 [40,41]. Class C fly ash was provided by China Power and Light Co., Ltd. (Hong Kong, China). Table 1 presents the chemical compositions of both the cement and fly ash, as determined by X-ray fluorescence analysis. Fine aggregate consisted of siliceous sand with a particle size ranging from 0 to 5 mm, while crushed granite stone of 5–10 mm served as the coarse aggregate. To ensure adequate workability of the mixture at a low water-to-binder ratio, Point-S polycarboxylate ether superplasticizer (manufactured by Kezhijie Co., Ltd., Xiamen, China), which offers a 30% water reduction, was added to adjust the rheological properties. Copper-plated hooked-end steel microfibers, measuring 14 mm in length and 0.22 mm in diameter (Figure 1), were supplied by Shanghai Zhenqiang Fiber Co., Ltd. (Shanghai, China). Thesemicro-fiberss possess a tensile strength of approximately 3000 MPa and an elastic modulus of about 200 GPa. The selection of 14 mm fiber length was based on the following considerations: (a) dispersion—short fibers are more easily and uniformly dispersed at low dosages (0.2–0.4 vol.%) without balling, which is particularly important for maintaining SCC flowability; (b) size compatibility—with the maximum coarse aggregate size of 10 mm, the 14 mm fibers (fiber-to-aggregate size ratio ≈ 1.4) can effectively bridge across aggregate gaps and arrest micro-cracks at their initiation sites; (c) cost-effectiveness—short fibers are less expensive than longer fibers and require lower dosage to achieve adequate bridging, aligning with the “low-cost, high-performance” objective; and (d) research focus—this study targets crack-bridging and toughening functions at low fiber dosages, rather than structural-level reinforcement, for which short fibers are sufficient.

This study adopted Grade 50 structural concrete as the target grade. To avoid construction delays for the concrete railing, a 7-day compressive strength target exceeding 40.0 MPa was established, while a 28-day target of 58.5 MPa was set to achieve comparable strength levels. Table 2 presents the mix proportions for all concrete mixtures, all of which maintained a fixed water-to-binder ratio of 0.28. An HJW60 (Beijing Xinyu Luda Instrument Equipment Co., Ltd., Beijing, China) ordinary pan mixer was employed for concrete mixing. The SCC mixtures were placed into the greased steel molds in a single layer and allowed to consolidate under their own weight, without any vibration or compaction. The molds were positioned on a steel worktable during casting. After the surfaces were finished, polyethylene sheets were applied to the specimens to prevent moisture loss. After demolding, the samples underwent initial curing in a controlled environment at 25 ± 2 °C and 95 ± 5% relative humidity for 3 days, followed by air curing until the time of testing.

### 2.2. Specimens and Testing Procedures

Flowability tests were performed to evaluate the workability of SCC, following the Chinese national standard JGJ/T 283-2012 [42] (“Technical specification for application of self-compacting concrete”). The equipment used included a stainless steel open platform, a truncated conical cone, a hand trowel, and a ruler or measuring tape. The testing procedure involved the following steps: (1) placing fresh SCC slurry onto the wetted stainless steel open platform; (2) leveling the fresh concrete with a hand trowel; (3) lifting the cone vertically; (4) allowing the concrete to rest for 20–30 s, then measuring two mutually perpendicular diameters, denoted as d_1_ and d_2_ (in mm); and (5) calculating the average of these two diameters as the slump flow value. It should be noted that, according to JGJ/T 283-2012, comprehensive characterization of SCC workability also requires assessment of passing ability and segregation resistance. In this study, slump flow was used as the primary control indicator to ensure that all mixtures met the minimum filling requirement for the specific application of concrete railings, where reinforcement spacing is relatively large and passing ability requirements are less stringent. However, the absence of passing ability and segregation resistance data is acknowledged as a limitation of this investigation.

Compressive strength tests were conducted on six cube specimens (100 mm × 100 mm × 100 mm) per mix from Table 2, following the Chinese standard GB/T 50081-2019 [43] “Ordinary concrete mechanics performance testing standards.” An automatic compression testing machine applied a loading rate of 0.3 MPa/s to perform the tests. Measurements were taken for each mix at 3, 7, 28, and 90 days, with six specimens tested at each curing age for each mixture, totaling 24 specimens per mixture. The compressive strength was calculated using the following formula:

(1) $$R_{c}=\frac{N}{A}\times 0.95$$

In this equation, *R_c_* represents the compressive strength of the concrete cube specimen (MPa), *N* denotes the maximum load at failure (N), and *A* is the contact area of the cube specimen (mm^2^).

Flexural strength tests were carried out on three prismatic specimens measuring 400 mm in length, 100 mm in width, and 100 mm in height. A four-point bending configuration with a support span of 300 mm was adopted, following the same procedure described in references [14,44]. The tests were performed using a servo-hydraulic MTS 810 testing system (MTS Systems Corporation, Eden Prairie, MN, USA) operating in displacement control mode, with a loading rate of 0.05 mm/min.

Each flexural strength value was computed using the formula below:

(2) $$F_{l}=\frac{PL}{{bh}^{2}}$$

In this equation, *F_l_* represents the flexural strength (MPa), *P* is the maximum load at failure (N), *L* denotes the support span (mm), while b and h correspond to the width and height of the specimen (mm), respectively.

For dry shrinkage measurements, three prismatic beams (400 mm × 100 mm × 100 mm) were cast for each concrete mix as listed in Table 2. A micrometer gauge was employed, and the tests followed the “JTG 3420-2020 [45]” standard (highway engineering cement and concrete testing method). The drying shrinkage value (*ε*) was calculated as Equation (3). The results are reported as dimensionless values multiplied by 10^−4^, with the shrinkage strain expressed as ×10^−4^ to facilitate presentation.

ε = (*L*_0_ − *L_t_*)/*L*_0_ × 100% (3)

where *L*_0_ is the initial length of the specimen immediately after demolding, and *L_t_* is the length after t days of drying.

A concrete rapid freeze–thaw machine was employed to perform freeze–thaw cycle tests according to the Chinese standard GB/T 50082-2024 [46], which specifies the test methods for long-term performance and durability of ordinary concrete. The apparatus comprises four main components: freeze–thaw cycling, intelligent chip control, setting, and display. Its operating temperature spans from −25 °C to 25 °C, with an accuracy of ±0.5 °C. Per the specifications of GB/T 50082-2024, specimens were removed from the curing room following 24 days of standard curing and subsequently soaked in water for a period of 4 days before testing. During immersion, the water temperature was maintained at 20 ± 2 °C, and the water level was kept 25 mm above the concrete surface. Throughout the freeze–thaw process, the temperature at the center of the specimens was controlled between a maximum of 5 °C and a minimum of −18 °C. Each complete freeze–thaw cycle lasted 4–6 h. One cycle was defined as the specimen core temperature dropping from 5 °C to −18 °C and then rising back from −18 °C to 5 °C. Furthermore, the thawing period was required to occupy no less than one-quarter of the total cycle time, and the transition time between freezing and thawing could not exceed 20 min. At specific intervals of 0, 25, 50, 75, 100, 150, and 200 cycles, concrete specimens were taken out for measurements of mass loss and compressive strength reduction. Each test group consisted of three specimens.

To evaluate the frost resistance of SCC with different steel fibers, this paper first investigates the mass loss rate with different numbers of freeze–thaw cycles. Upon reaching 28 days of curing, the specimens were removed, and their initial mass (M0) was recorded. Subsequently, following the experimental schedule, the masses of specimens subjected to various numbers of freeze–thaw cycles were measured as Mn. The mass loss rate after freeze–thaw cycling was then calculated using Equation (4).

(4) $$∆M=\frac{\;M_{o}-M_{n}\;}{\;M_{o}}\times 100\%$$

In this equation, *ΔM* is the percentage mass loss of the concrete specimen following n freeze–thaw cycles, with *M*_0_ representing the original specimen mass (g) and *M_n_* indicating the specimen mass after n cycles (g).

Additionally, a DT-20 dynamic elastic modulus tester was employed to evaluate concrete specimens at various stages of freeze–thaw cycling, and the measured data were subsequently used to determine the relative dynamic elastic modulus. The testing procedure consisted of the following steps: 1. The mass and dimensions of each concrete specimen (100 mm × 100 mm × 400 mm) were recorded. Mass measurements were accurate to within ±0.5%, while dimensional measurements were precise to within ±1%. The cross-sectional dimensions and length of each specimen were determined by averaging values taken from three different locations. 2. The specimen was placed on a foam pad to keep it from shifting. The transmitting probe was situated at the side’s midpoint, 200 mm away from the specimen’s edge, while the receiving probe was placed 5 mm from the edge. 3. Three consecutive readings were taken per measurement. If the spread among these readings was within 0.5%, the value was recorded. Two measuring points per side resulted in four points per specimen. The final test result for each specimen was taken as the average of these 12 individual readings (4 points × 3 repetitions).

The dynamic elastic modulus (E) of specimens was calculated through Equation (5):

(5) $$E=\frac{13.244\times 10^{-4}\times M\times 400\times 10^{3}F^{2}}{100\times 10^{4}}$$

where *E* represents the dynamic elastic modulus (GPa), *M* is the mass of the concrete specimen (kg), and *F* denotes the frequency measured by the dynamic elastic modulus tester (Hz).

In evaluating the frost resistance durability of concrete, the relative dynamic elastic modulus (*∆E*) serves as the durability indicator. The *∆E* values for all specimens after freeze–thaw cycling were calculated using Equation (6):

(6) $$∆E=\frac{E_{n}}{E_{0}}\times 100\%$$

where *∆E* is the relative dynamic elastic modulus of the concrete specimen after a given number of freeze–thaw cycles (%), *E*_0_ is the initial dynamic elastic modulus of the specimen (GPa), and *E_n_* is the dynamic elastic modulus after n freeze–thaw cycles (GPa).

Porosity and pore size distribution were measured using an AUTOSCA-V 9600 Mercury Intrusion Porosimeter (MIP) (Micromeritics Instrument Corp., Norcross, GA, USA), capable of detecting pores ranging from 10 nm to 200 μm. A contact angle of 140° and a mercury surface tension of 480.0 erg/cm^2^ were applied. Prior to testing, the samples were dried in an oven at 105 ± 2 °C for 24 h.

All tests were performed in triplicate for each concrete mixture (n = 3 per group). Results are reported as mean ± standard deviation (SD). The SD was calculated from the three parallel measurements for each group at each testing age. Statistical analysis was performed using two-way analysis of variance (ANOVA) to evaluate the significance of the effects of two independent variables, namely steel fiber volume fraction and superplasticizer (SP) dosage, as well as their interaction, on the measured properties (drying shrinkage and freeze–thaw mass loss). A significance level of *p* < 0.05 was considered statistically significant, with *p* < 0.01 and *p* < 0.001 denoted as highly significant.

## 3. Results and Discussion

### 3.1. Flowability of Fresh SCC

**Figure 2:**
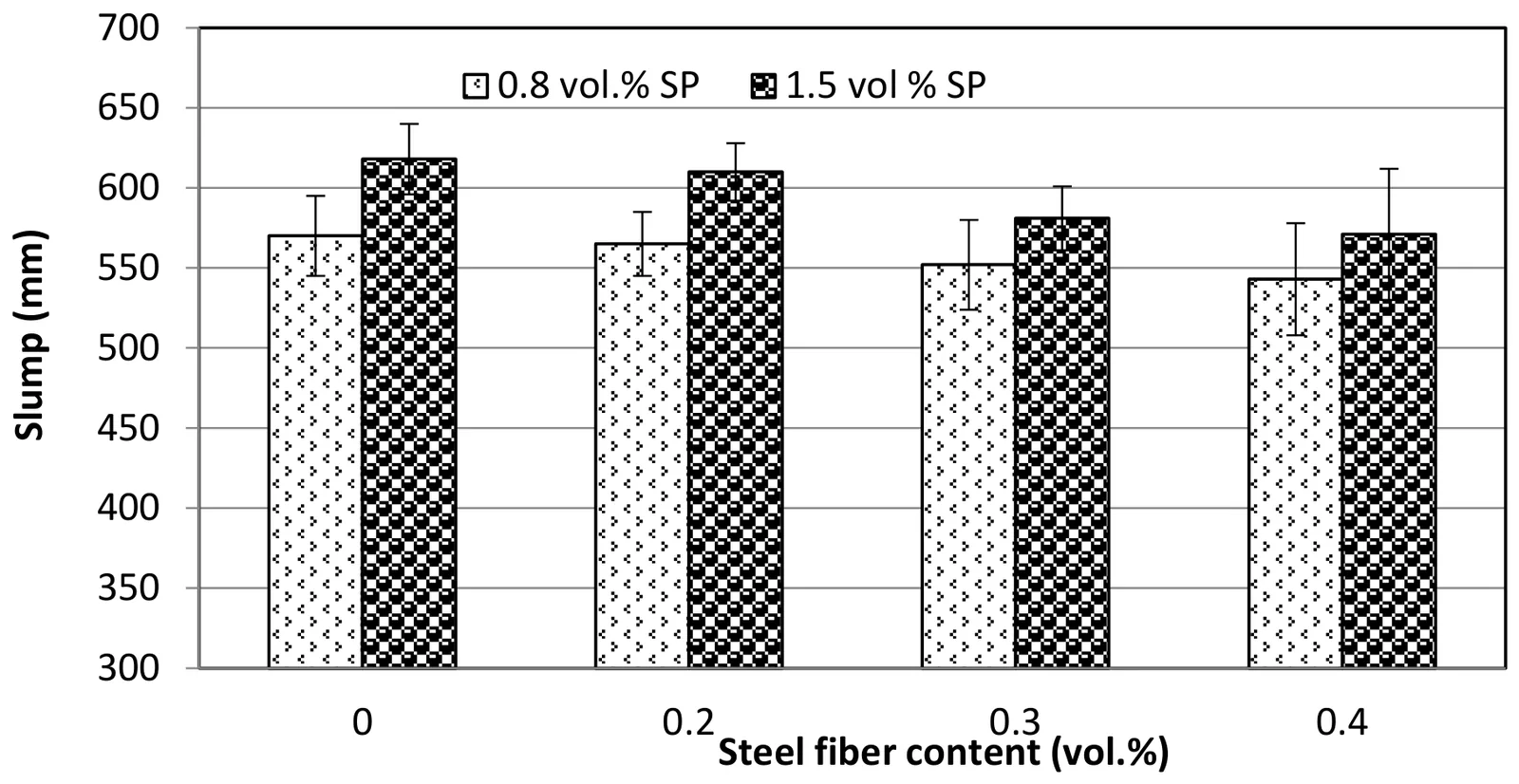
Slump test results with various SP and steel fibers.

Figure 2 illustrates the effect of steel fibers on the flowability of SCC with different SP content. This figure shows that, for the two series of concrete, the flowability of SCC decreases with the increase in steel fiber content. This decrease is about 1.2~6.1% compared to control samples without steel fibers. This result is similar to other research results where steel fibers with a fixed w/c ratio were used in the concrete mixture [22]. As reported by R. Yu et al., the relative slump value sharply dropped from 4.29 to 1.10 with an increase in the steel fiber content from 0.5 vol.% to 2.5 vol.% [47]. Similarly, Su-Jin Lee et al. [25] reported that the incorporation of 0.47 vol.% (37 kg/m^3^) of 35 mm-length steel fiber (aspect ratio: 64) reduced the slump flow of the fiber-reinforced mixtures by 51.2% as compared to the blank one. The negative effect of steel fibers on the workability of concrete is mainly due to the following three reasons [47]: (a) steel fibers possess a much more elongated shape than aggregates, and their surface area per unit volume is larger, which enhances the cohesive forces at the fiber–matrix interface. (b) Rigid fibers alter the granular skeleton structure by displacing particles that are large relative to the fiber length. (c) Steel fibers are frequently manufactured with deformations (such as hooked ends or wavy shapes) to improve anchorage with the surrounding matrix. The friction between hooked-end steel fibers and aggregates is higher compared with straight steel fibers [47]. In this study, short and hooked-end steel fibers are used, which means the workability loss of concrete with the addition of fibers should be attributed to: (1) the increase in the internal surface area that produces higher cohesive forces between the fibers and concrete matrix and (2) the anchorage effect of hooked ends that increase the friction in SCC.

Considering the effect of SP, as shown in Figure 2, test results show that SP has a positive effect on the fresh behavior of SCC, as expected, and the more the SP content, the higher the flowability will be. As shown in Figure 2, with the same content of steel fibers, the slump of Series II concrete is about 5.1~8.4% higher than that of Series I. Additionally, it is worth noting that the flowability of all SCC samples except for SCC08/4 meets the target workability of SCC, i.e., 550 mm for slump, according to the National Standard of China JGJ/T 283-2012.

### 3.2. Compressive Strength

**Figure 3:**
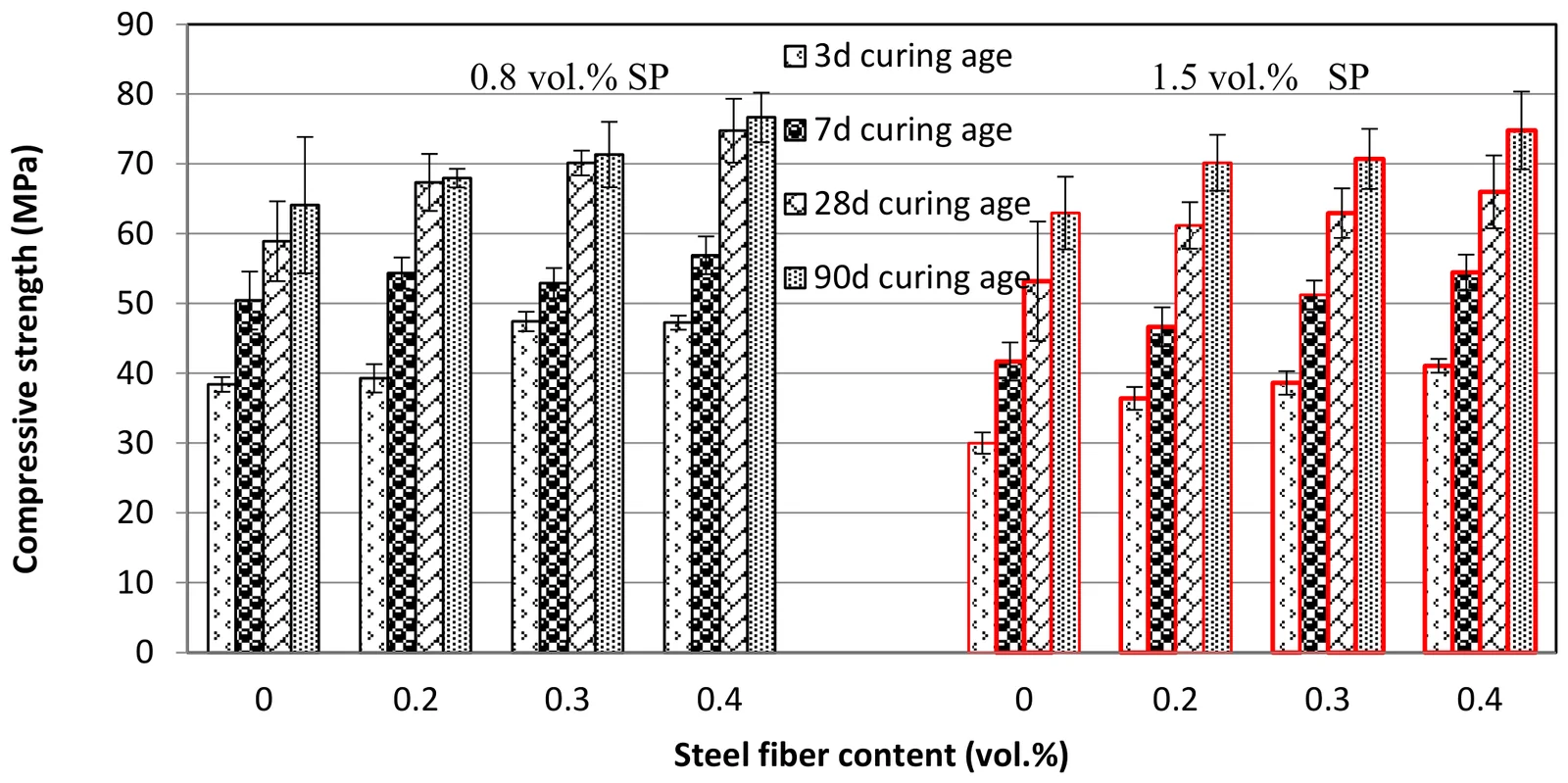
Values of compressive strength for all mixes.

The reinforcement effect of steel fibers on the compressive strength of SCC after 3 days, 7 days, 28 days, and 90 days of curing is shown in Figure 3. It is obvious that the compressive strength of all concretes except for SCC15/0 meets the target of Grade 50 railing concrete, i.e., more than 40.0 MPa for 7-day strength and 58.5 MPa for 28-day strength, respectively. Moreover, Figure 3 shows that the addition of steel fibers generally increased the compressive strengths of SCC similarly as reported by other researchers [7,35,48,49]. The increase in compressive strength is due to the increase in density and rigidity in the presence of steel fibers, as well as enhancement of the restraining action of fibers to the propagation of micro-cracks before reaching the ultimate strength [48,50,51]. For Series I mixtures, as shown in Figure 3, SCC08/4 had the highest 28-day strength of 74.7 MPa, followed by SCC080.4% having a 28-day strength of 70.1 MPa, and SCC08/2 having a 28-day strength of 67.4 MPa, which was about 14% higher than that of SCC080. Additionally, Figure 3 shows that the compressive strengths decreased with the increase in SP content, and the 3-day, 7-day, 28-day, and 90-day strengths of Series II concretes containing 1.5% SP were about 7.4~20.1%, 3.1~17.3%, 6.4~9.7%, and 0.8~3.2% lower than that of Series I concretes containing 0.8% of SP, respectively. The disadvantageous effect of SP on the compressive strength of SCC was also obtained by H. Yılmaz Aruntaş [52], when 28-day and 90-day strengths were considered, the maximum strengths were obtained with 1.0% SP dosage as compared to that with 1.5% and 2.0% SP. Similarly, P. Basu et al. found that the increase in SP content from 1.3% to 1.8% reduced the 28-day compressive strength of SCC from 50.5 MPa to 38.33 MPa with a water-binder of 0.34 [36]. M. Benaich et al. also found that the compressive strength of SCC generally decreased with increasing of SP dosage, and specially, the increasing of SP dosage from 0.3% to 1.0% reduced the 1-day, 7-day and 28-day compressive strength of SCC by 30 MPa (from 45.2 MPa to 15.04 Mpa), 40 MPa (from 60.8 MPa to 20.76 Mpa) and 44 MPa (from73.48 MPa to 29.44 Mpa), respectively [36,37]. This reduction in compressive strength may be caused by the entraining air bubbles under higher SP dosage in the fibrous concrete [52].

**Figure 4:**
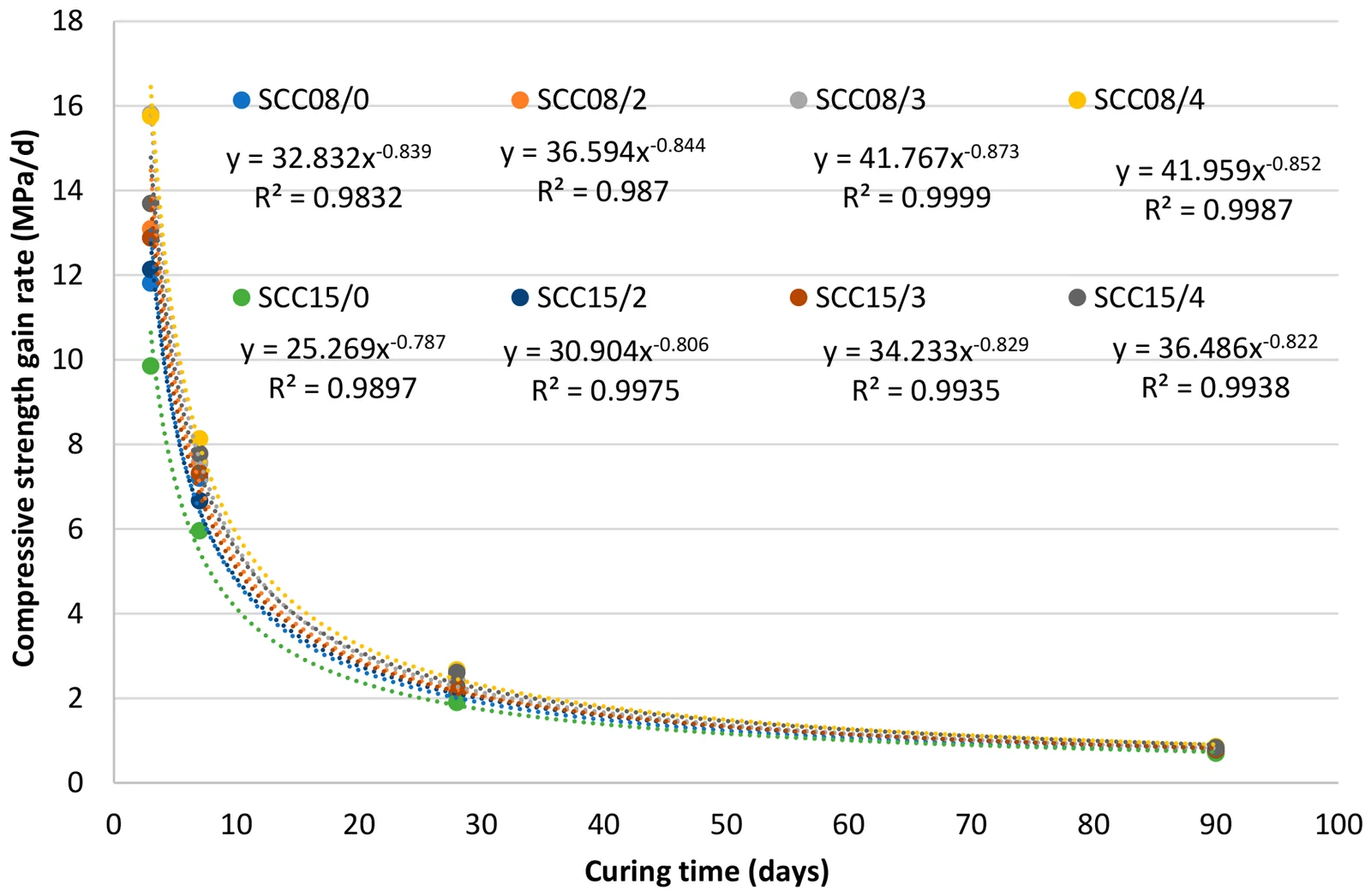
Compressive strength development rate ($\overset{⃑}{V_{R}}$) for all mixes after 90 days of curing.

To better understand the effect of steel fibers and SP content on the compressive strength development of SCC after 3~90 days of air curing, the compressive strength gain rate $\overset{⃑}{V_{R}}$ was calculated by using Equation (7), and the results are shown in Figure 4.

(7) $$\overset{⃑}{V_{R}}=\frac{R_{t}}{t_{c}}$$

where $\overset{⃑}{V_{R}}$ is the strength gain rate of concrete (MPa/d), *R_t_* is the average strength of SCC after “*t*” days of curing (MPa), and “*t_c_*” is the curing time (days).

**Figure 5:**
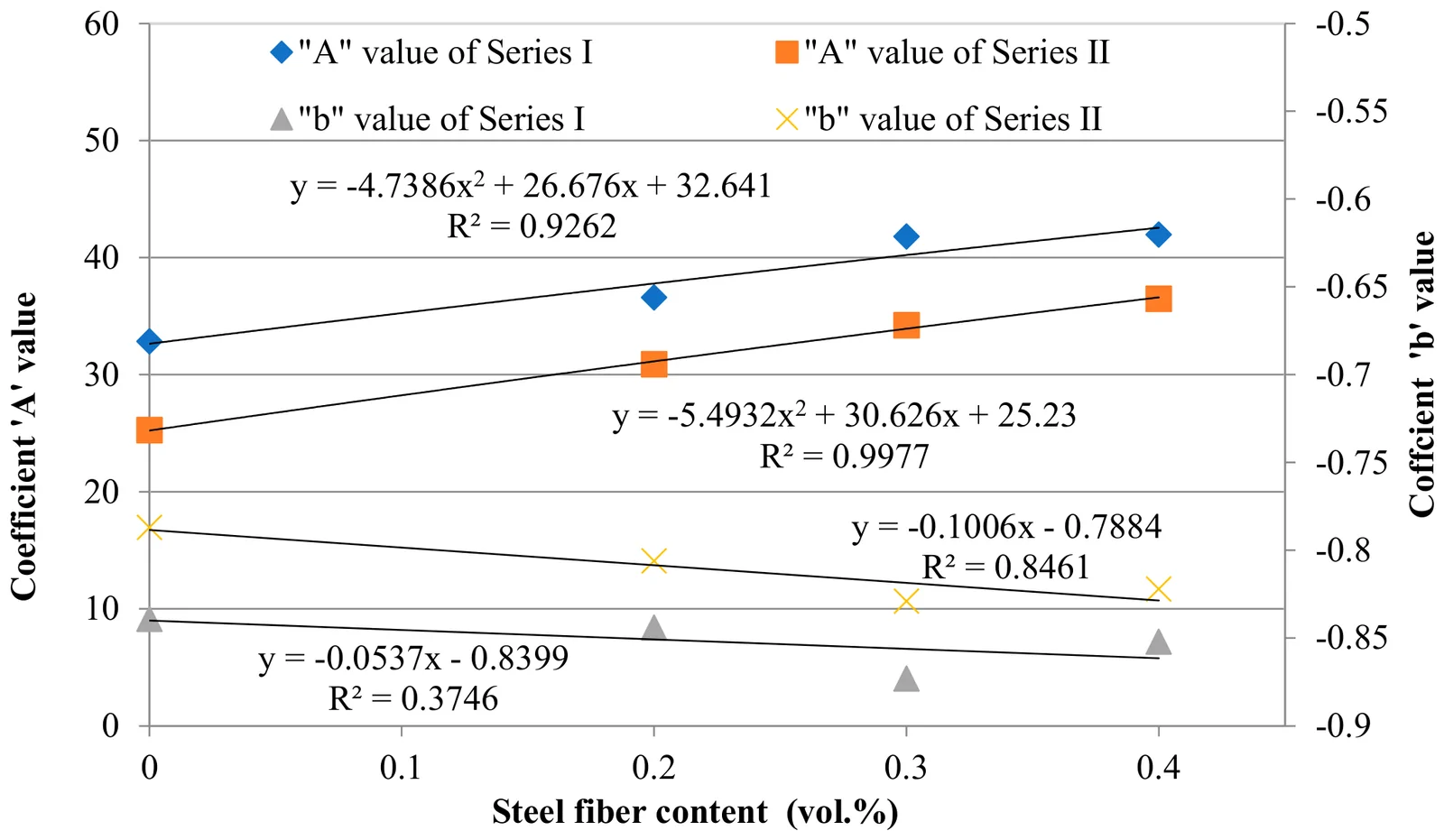
Coefficient “A” and “b” values of all mixes.

As shown in Figure 4, the compressive strength of all concretes developed rapidly at early curing ages as expected, and the incremental value of $\overset{⃑}{V_{R}}$ between 3 and 28 days was generally higher than that between 28 and 90 days. Moreover, Figure 4 shows that the relationship between $\;\overset{⃑}{V_{R}}$ and curing time (t) followed the negative power function model, with coefficients of determination (R^2^) close to 1, indicating a strong correlation between $\overset{⃑}{V_{R}}$ and curing time. Accordingly, the relationship between $\overset{⃑}{V_{R}}$ and “t” could be characterized by using Equation (8):

(8) $$\overset{⃑}{V_{R}}=At^{b}$$

where $\overset{⃑}{V_{R}}$ is the compressive strength gain rate (MPa/d) of SCC, *t* is the curing age (days), and coefficients *A* and *b* depend on the mix proportion, as shown in Figure 5.

As shown in Figure 5, the coefficient A exhibited a quadratic relationship with steel fiber content within the experimental range (0–0.4 vol.%), with R^2^ values close to 1 for both series. This empirical correlation provides a convenient means to estimate the strength gain rate for a given fiber content within the tested range. However, it is emphasized that this quadratic fitting is strictly valid only within the experimental domain and should not be extrapolated beyond the tested fiber content range.

From Figure 5, the fitting formula for Series I is:

(9) $$f\left(x\right)=-4.7386x^{2}+26.676x+32.641$$

where “x” is the content of steel fibers.

Similarly, from Figure 5, we see the fitting equation of Series II

(10) $$f\left(x\right)=-5.4932x^{2}+30.626x+25.23$$

It is worth noting that, in both series, the quadratic fitting yields theoretical maximum values at fiber contents far beyond the experimental range (approximately 2.8 vol.%). Such extrapolated values are not considered valid in this study, as they lie well outside the tested domain. For reference, previous studies [48,49,53,54] have reported optimum steel fiber contents in the range of 2.0–3.0 vol.% for conventional concrete; however, these high fiber contents are not applicable to SCC due to the severe workability loss they would cause. The present study focuses exclusively on low-volume steel fiber-reinforced SCC (0–0.4 vol.%), within which all mixtures were designed to maintain self-compacting performance.

In addition, Figure 5 shows that, at a given steel fiber content, increasing the SP dosage decreased the A value, as the curve of Series II consistently fell below that of Series I within the experimental range. This indicates that excessive SP (1.5 wt.% in this study) may have a negative effect on the compressive strength development of SCC. The underlying mechanism warrants systematic investigation in future work.

Regarding the coefficient b in Equation (8), as shown in Figure 5, its mean value was approximately 0.83 ± 0.043. The correlation between the b value and steel fiber content was weak, with R^2^ values of only 0.3746 and 0.8414 for Series I and II, respectively, suggesting that b is relatively insensitive to fiber content within the tested range.

### 3.3. Flexural Strength

**Figure 6:**
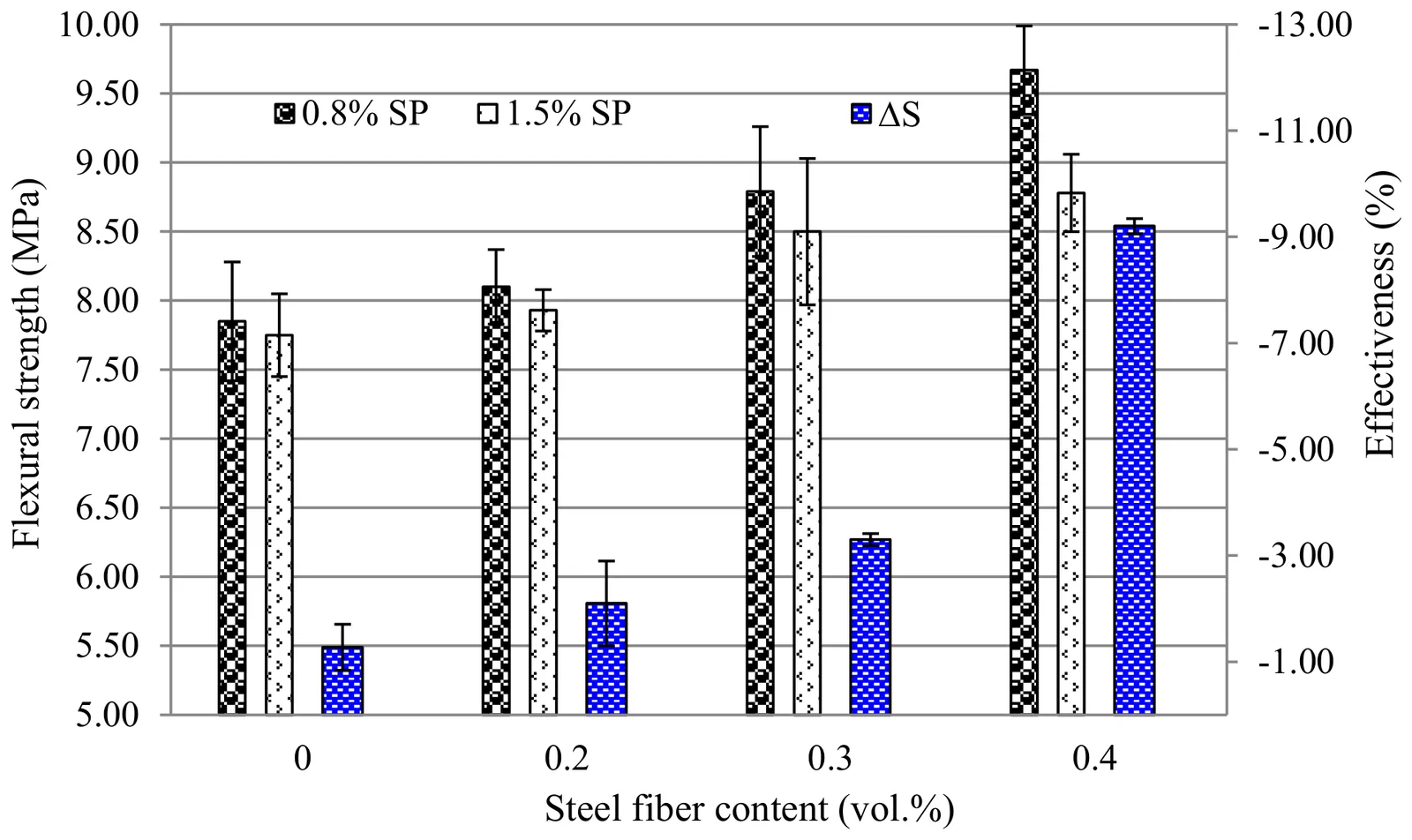
Flexural strength of SCC with different SP and steel fiber content.

Flexural strength serves as a critical indicator for assessing the tensile performance of fiber-reinforced cementitious composites. A higher flexural strength typically corresponds to greater ductility. The obtained four-point bending test results for flexural strength are shown in Figure 6. Clearly, the flexural strength values of the specimens without steel fibers for Series I and Series II were measured as 7.0 MPa and 6.8 MPa, respectively. It was observed in Figure 6 that the increase in steel fiber content led to an increase in the flexural strength values. For all test samples, the highest flexural strength value was obtained in the Series I mixtures with 0.4 vol.% of steel fiber, which was about 38.1% higher than that of SCC08/0 without fibers. Moreover, Figure 6 shows that the lowest flexural strength value was obtained from Series II mixtures including 1.5% SP and 0% steel fibers. In addition, it is worth noting that the incorporation of 0.2 vol.% steel microfibers could increase the flexural strength by about 16~17%, indicating that a low volume of microfibers is effective in transferring stress and controlling cracks [38,39]. Also, Figure 6 shows that the flexural strength values of Series I were generally higher than those of Series II, and the ∆S value decreased with the increase in steel fibers. This result illustrates that the higher SP dosage had a negative effect on the efficiency of steel fiber in transferring stress and controlling cracks; the reason may be due to the air pores entrained by SP, which weaken the interfacial bonding force between steel fibers and cement matrix.

### 3.4. Brittleness Index

**Figure 7:**
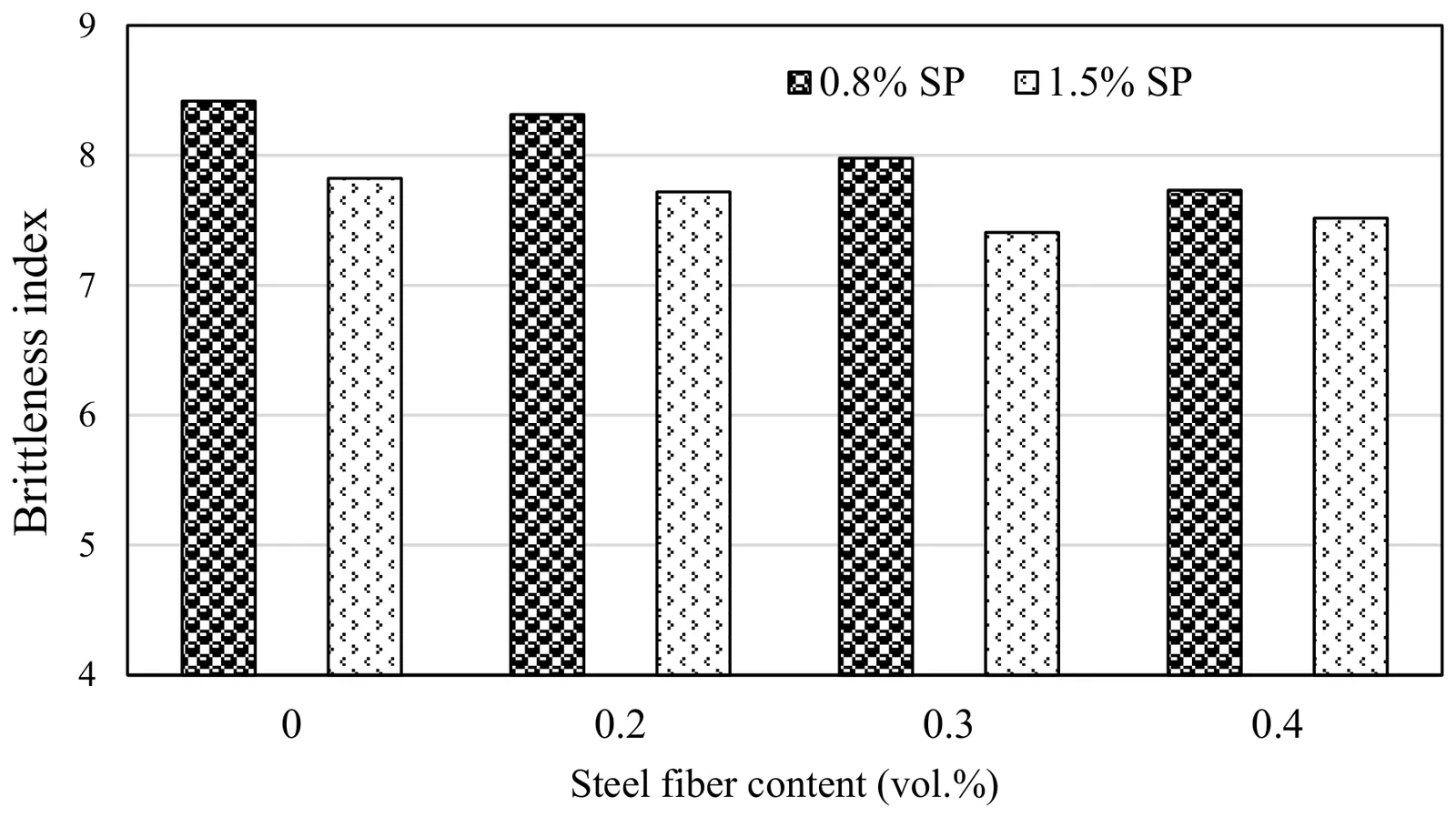
Brittleness index of SCC with different SP and fiber content.

The brittleness index (BI) is defined as the ratio of compressive strength to flexural strength [55]. A higher BI value indicates greater brittleness of the specimen. Figure 7 presents the 28-day BI values calculated for each sample in this study. Higher BI values are associated with SCC08/0 and SCC15/0 mixtures. A notable decline in BI value is observed with the increase in steel microfibers, indicating the enhancement effect of fibers on toughness. Moreover, Series II has lower BI values than Series I. This is likely because the higher SP dosage increases the shape-shifting ability under compressive stress, a mechanism similar to the observation that low-strength concretes are tougher than high-strength concretes.

### 3.5. Dry Shrinkage Performance

**Figure 8:**
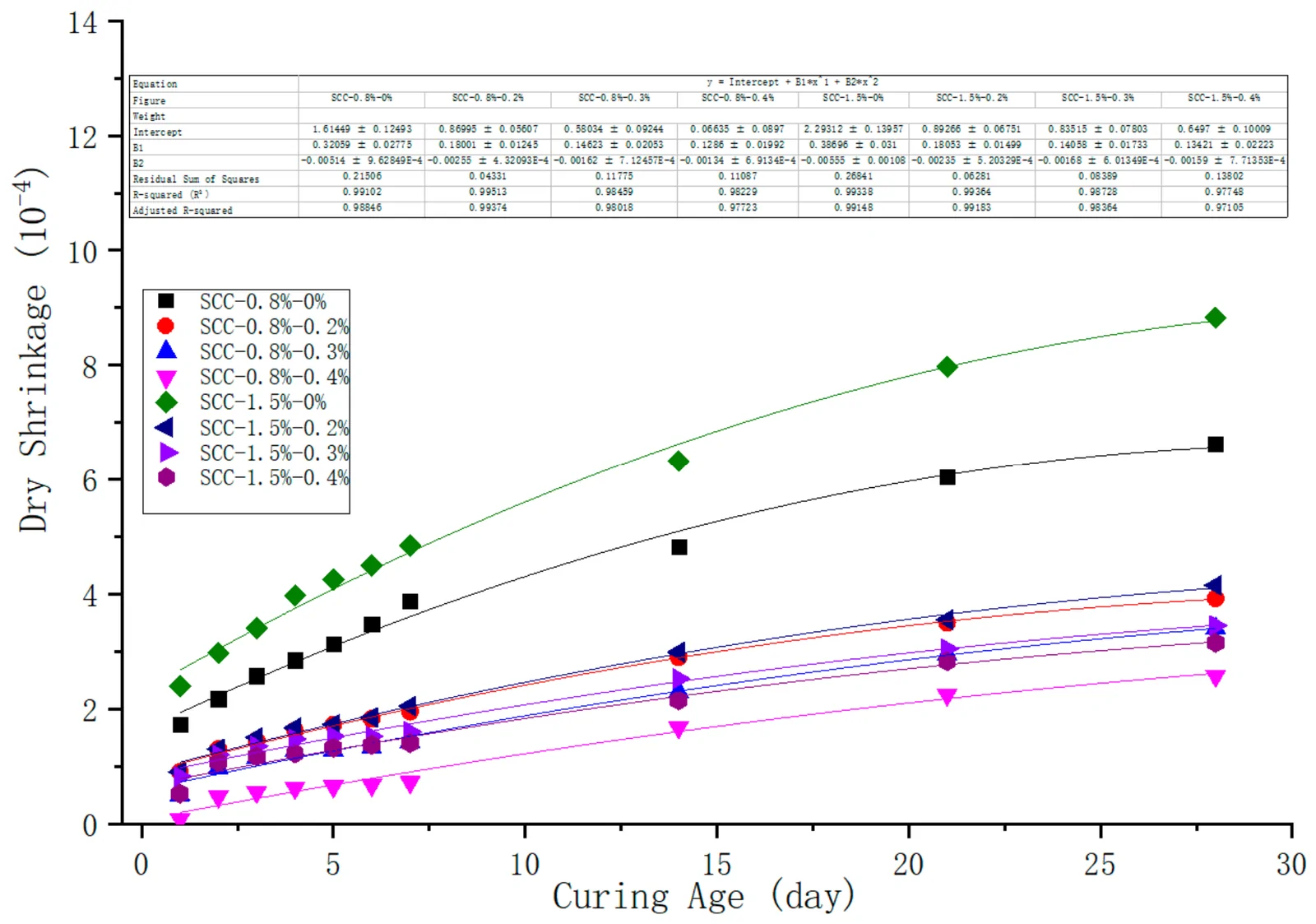
Dry shrinkage results.

Fibers can mitigate drying shrinkage in concrete through two main mechanisms. First, they enhance the bond strength at the fiber–matrix interface, which physically restrains shrinkage [56]. Second, they control cracking—considered the most significant role of fibers in relation to concrete shrinkage [57,58,59]. The results of drying shrinkage tests for SCC with low-volume steel microfibers are shown in Figure 8. The findings indicate that the incorporation of steel fibers at any dosage led to a considerable reduction in drying shrinkage of the concrete. As illustrated in Figure 8, higher fiber volume fractions resulted in greater shrinkage reduction. Specifically, compared to the reference mix SCC08/0, the addition of 0.2~0.4 vol.% steel fibers reduced drying shrinkage by 40~61% for Series I concrete and by 37~52% for Series II concrete. Additionally, Figure 8 shows that the lowest drying shrinkage was achieved in the mixtures containing 0.4 vol% fibers, followed by the incorporation of 0.3 vol.% steel fibers, and then by the addition of 0.2 vol.% fibers. Even so, the dry shrinkage of concrete having 0.2 vol% fibers was about 41~53% lower than that of the plain one. The reduction in drying shrinkage observed in this study is higher than that reported in previous studies [57,58,60]. These results demonstrate that hooked-end steel microfibers represent a viable solution for controlling concrete drying shrinkage.

Considering the effect of SP, as shown in Figure 8, regardless of steel fiber content, the drying shrinkage of concrete increased with an increase in SP content, and increasing SP dosage from 0.8% to 1.5% increased the drying shrinkage by 15% to 74%. The increase in drying shrinkage may be related to the increase in capillary pores of concrete caused by increasing SP content. It is widely recognized that drying shrinkage in concrete primarily results from water loss in capillary pores, which is driven by capillary tension between water and the pore walls [61]. Therefore, mercury intrusion porosimetry (MIP) was employed to further investigate the porosity and pore size distribution of the tested specimens. Statistical analysis using two-way ANOVA further confirmed that fiber volume fraction (*p* < 0.001), SP dosage (*p* < 0.001), and their interaction (*p* = 0.023) all exerted significant effects on drying shrinkage at 28 days.

### 3.6. Freeze–Thaw Resistance

**Figure 9:**
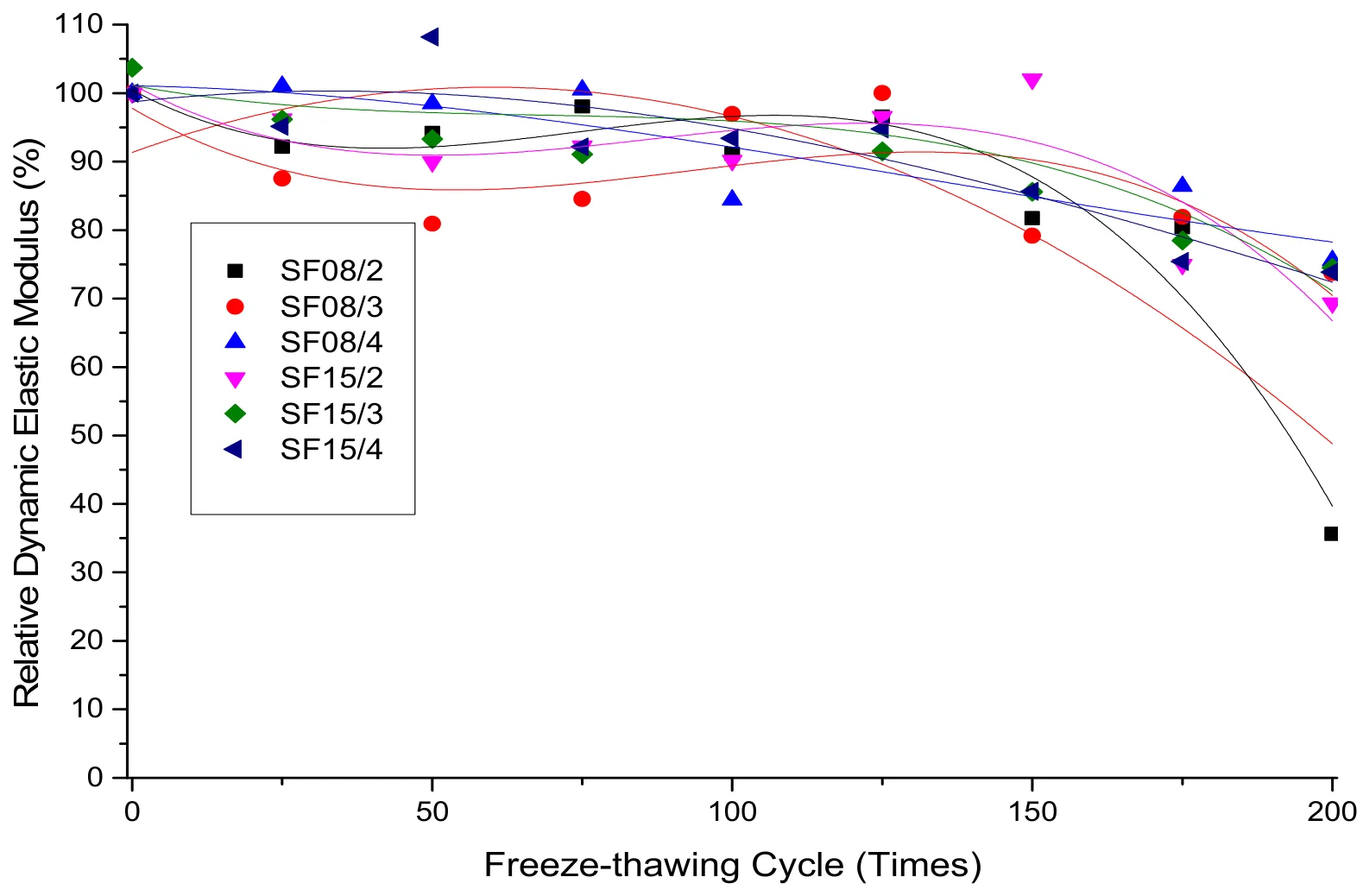
Relative dynamic modulus during the freeze–thaw cycles.

Many railing structures are situated in cold environments, where they are subjected to freeze–thaw cycles. Some have even sustained severe damage during their design service life, rendering them unable to function properly. To quantitatively assess the freeze–thaw-induced deterioration of the specimens, the average rate of change in the relative dynamic elastic modulus (RDEM) was employed as the key indicator. Figure 9 illustrates the complete evolution of RDEM for each specimen. Examining the trends in Figure 9, it can be observed that RDEM loss remains relatively insignificant during the early stages. However, when the number of freeze–thaw cycles reaches approximately 100 to 150, a decline in RDEM occurs in all samples except for SCC08/4. This indicates that freeze–thaw action begins to exert a noticeable impact on concrete once a critical threshold is reached. In addition, Figure 9 shows that, for all mixtures, SCC08/4 comprising 0.4 vol% steel fibers had the greatest RDEM, and the 200 freeze–thaw cycles only reduced the RDEM value by 24.4%. This was followed by SCC15/4, SCC15/3, and SCC08/3, whose RDEM values were about 1.1~1.8% lower than that of SCC08/4. The RDEM of SCC08/2 containing 0.2 vol.% steel fibers was identified as the lowest, and the 200 freeze–thaw cycles only reduced the RDEM value by 64.5%.

**Figure 10:**
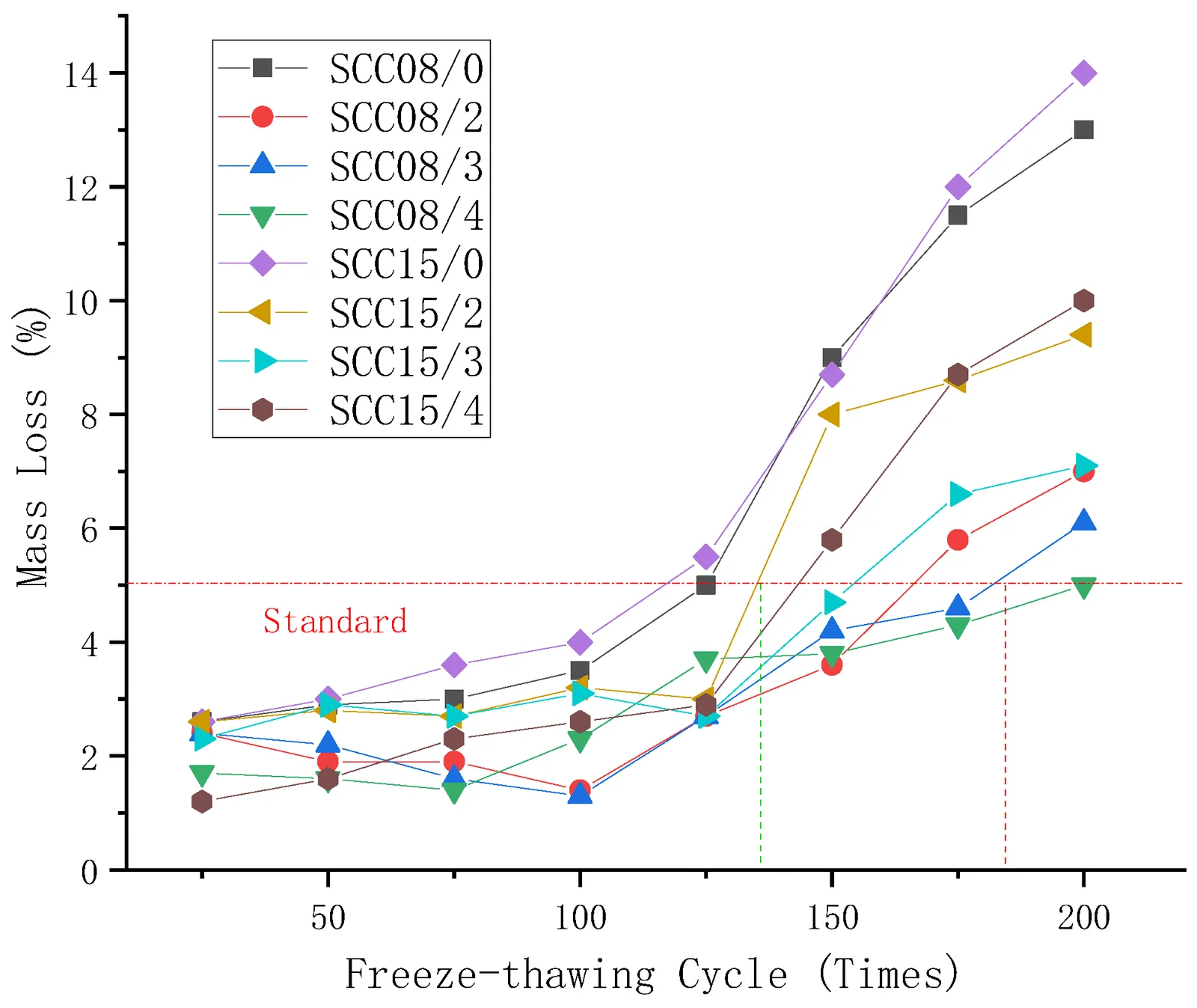
Mass loss during the freeze–thaw cycles.

The mass loss rate for each specimen was determined using Equation (4), based on mass measurements taken before and after freeze–thaw damage. Figure 10 presents the changes in mass loss observed in concrete specimens throughout the freeze–thaw cycling process. Overall, the mass loss of all mixtures remained relatively low and stable before 125 cycles, followed by a pronounced increase thereafter, indicating that freeze–thaw damage becomes dominant once a critical cumulative damage threshold is reached. Notably, the control groups without steel fibers (SCC08/0 and SCC15/0) exhibited the most severe deterioration, with mass losses of 13.0% and 14.0% after 200 cycles, respectively, far exceeding the 5.0% failure criterion specified in the “Test method for long-term performance and durability of concrete” (GB/T 50082-2009). This confirms that the incorporation of steel fibers significantly enhances the freeze–thaw resistance of the concrete. Among the fiber-reinforced groups, the frost resistance varied markedly depending on the SP dosage. The SCC08/4 mixture demonstrated the best overall performance, with a mass loss of only 5.0% after 200 cycles, falling just at the failure threshold. In comparison, the SCC08/3 and SCC15/2 mixtures showed moderate durability, with mass losses of 6.1% and 9.4% at 200 cycles, respectively. Notably, the SCC15/2 mixture exceeded the 5.0% limit before 150 cycles (reaching 8.0% at 150 cycles), indicating poor frost resistance. However, the SCC15/4 and SCC08/2 groups exhibited poor performance, with the SCC15/2 mixture already exceeding the 5.0% limit before 150 cycles (reaching 8.0% at 150 cycles), indicating that excessive SP content combined with high fiber volume may adversely affect the matrix microstructure and reduce frost resistance. Statistical analysis using two-way ANOVA further confirmed that fiber volume fraction (*p* < 0.001), SP dosage (*p* < 0.001), and their interaction (*p* < 0.001) all exerted highly significant effects on freeze–thaw mass loss at 200 cycles.

According to the above RDEM and mass loss test results, it is evident that steel fibers had a positive effect in improving the freezing and thawing resistance of SCC, and within the tested range, the higher the fiber content, the stronger the resistance. However, the higher SP dosage had a negative influence on the freeze–thaw reliability, as Series II generally had higher mass loss and lower RDEM than Series I. Moreover, it is worth noting that, when flowability, cost, and durability were considered, SCC08/3 showed acceptable performance before 175 freeze–thaw cycles (RDEM: 19.1%; mass loss: 4.6%), but its mass loss exceeded the 5% failure limit at 200 cycles (6.1%). Therefore, for severe freeze–thaw environments, only SCC08/4 is recommended; for milder conditions, SCC08/3 may serve as a cost-effective compromise.

### 3.7. Porosity and Pore Size Distribution

Porosity and pore size distribution are widely regarded as key factors governing both the durability and strength of cementitious materials. It is generally accepted that cementitious materials with higher porosity and larger pore sizes tend to exhibit inferior durability and lower strength. To examine how the addition of SP influences the porosity and pore size distribution of concrete, the results for mixes SCC08/3 and SCC15/3 were evaluated and are discussed in the following section.

**Figure 11:**
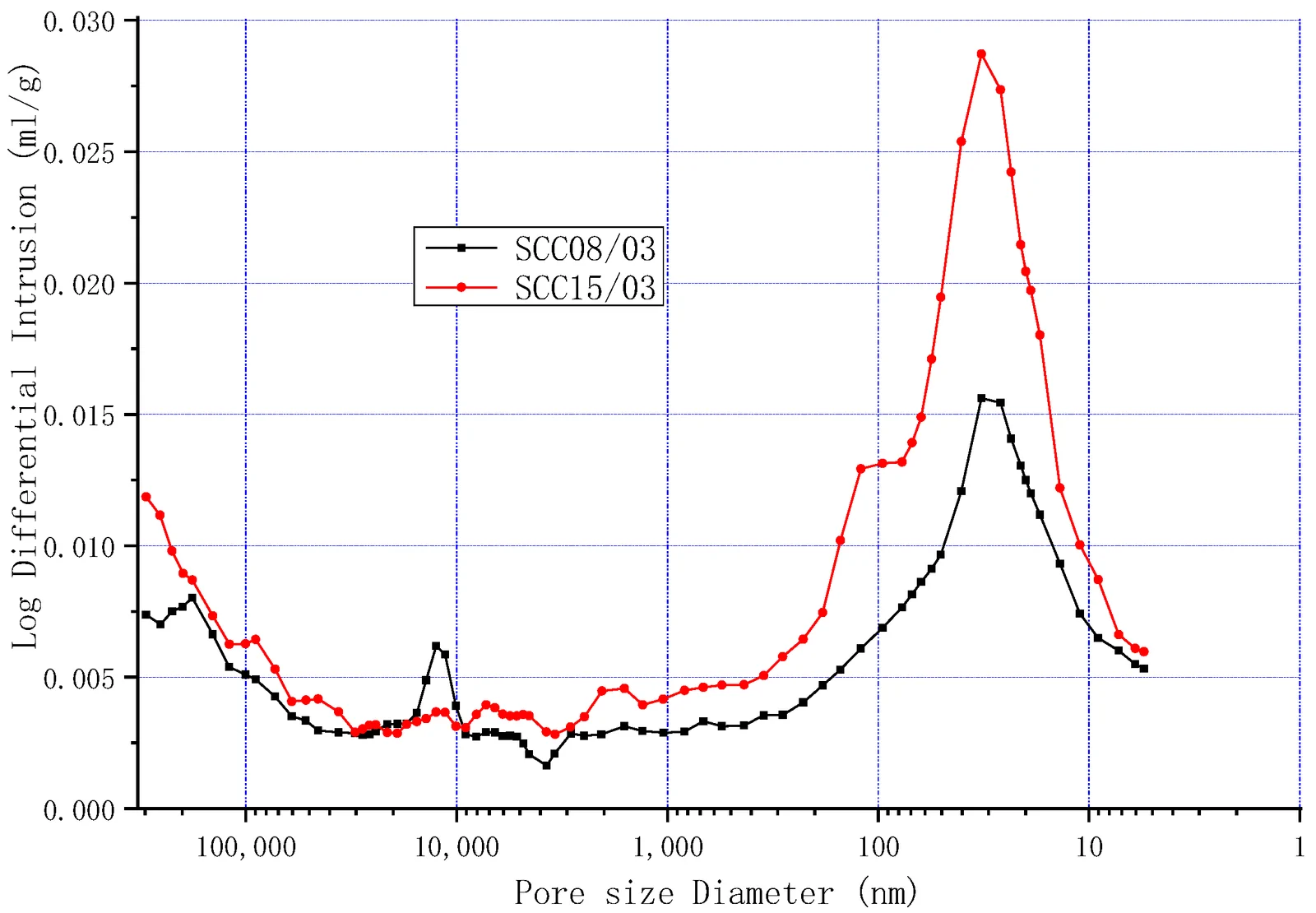
Typical pore size distribution at 28 days.

Table 3 summarizes the raw MIP test data for mixes SCC08/3 and SCC15/3 after 28 days of curing. It is evident that the addition of a high SP content significantly affected both porosity and pore size distribution. Compared to SCC08/3, the total porosity of SCC15/3 increased by up to 18%. According to Mehta and Monteiro, total porosity directly influences the physical properties of cement-based materials, including strength and durability. The higher porosity observed for SCC15/3 explains its lower strength values, as shown in Table 3 and Figure 11. Table 3 also reveals that increasing the SP content substantially raised the proportion of pores smaller than 50 nm, from 2.45% in SCC08/3 to 3.29% in SCC15/3. Chakraborty [62] noted that pores ≤ 50 nm primarily affect concrete creep and shrinkage, whereas pores ≥ 50 nm govern permeability and strength. The higher value of pores ≤ 50 nm for SCC15/3 was consistent with its higher drying shrinkage as presented in Figure 10.

Figure 11 illustrates the relationship between Log Differential Intrusion (LDI) and pore radius. For the SCC08/3 mixture, LDI increases as pore radius decreases, reaching a maximum of 0.016 at a radius of 20 nm. Below this radius, LDI progressively declines with further reductions in pore radius. The SCC15/3 mortar exhibits a similar trend, also peaking at 20 nm; however, its maximum LDI value is substantially higher than that of SCC08/3. Additionally, an extra peak appears at a much larger radius of approximately 150 nm. These observations suggest that incorporating an excessive amount of SP leads to a deterioration of the pore structure, characterized by both increased total porosity and an unfavorable shift in pore size distribution, as evidenced by the emergence of larger pores (50 nm) in the SCC matrix.

Based on the comprehensive experimental results presented above, a multi-criteria evaluation was performed to identify the optimal mixture for railing structure applications. The evaluation considered four key performance indicators: (1) workability (slump flow ≥ 550 mm); (2) mechanical properties (28-day compressive strength ≥ 53.2 MPa and flexural strength ≥ 7.8 MPa); (3) durability (mass loss after 125 freeze–thaw cycles ≤ 5.0%); and (4) cost-effectiveness (lower SP and fiber dosages are preferred). Among all tested mixtures, SCC08/3 (0.8% SP + 0.3 vol.% fibers) uniquely satisfies all requirements: it exhibits adequate slump flow (approximately 552 mm), high compressive strength (70.1 MPa), flexural strength (8.8 MPa), and acceptable freeze–thaw performance (mass loss of 6.1% at 200 cycles, close to the 5% threshold). Although SCC08/4 offers superior freeze–thaw resistance (5.0% mass loss), its marginal performance gain over SCC08/3 does not justify the additional fiber cost. Conversely, the 1.5% SP series (SCC15/x) consistently underperforms in strength and durability due to increased porosity, as confirmed by MIP analysis. Therefore, SCC08/3 is recommended as the optimal cost-effective mixture for practical railing construction. This recommendation is further supported by the two-way ANOVA results, which confirm that fiber volume fraction is the dominant factor controlling both shrinkage and freeze–thaw resistance, while SP dosage plays a significant but secondary role.

## 4. Conclusions

Quasi-brittle concrete railings are used along road sections and bridges to ensure safety. However, such railings are particularly susceptible to the initiation and propagation of cracks of various sizes within their structure. The addition of steel fibers could be a partial solution to alleviate the brittle behavior of concrete. In this study, the flowability, mechanical properties, and durability of self-compacting concretes containing two different superplasticizer dosages (0.8% and 1.5%) and a low volume of hooked steel microfibers (0~0.4 vol%) have been investigated through experimental research. Based on the test results, the following conclusions can be drawn:

1. The addition of superplasticizer enhanced the flowability of the concrete, whereas the inclusion of steel fibers had a minor adverse effect on its fresh properties. With the exception of SCC08/4 (0.8% SP + 0.4% fibers), all mixtures achieved the target workability specified for self-compacting concrete (SCC) by the Chinese national standard JGJ/T 283-2012, which requires a slump flow of 550 mm.
2. The incorporation of steel microfibers substantially enhanced both the strength and shrinkage resistance of SCC. In general, increasing the fiber content within the tested range (0–0.4 vol.%) led to improved mechanical performance and durability. However, higher SP dosage (1.5 wt.% in this study) is not recommended, as it increases the porosity of the composite and marginally compromises the mechanical properties.
3. The influence of curing age on compressive strength gain was considerably more pronounced during the early stages (3–28 days) than at later ages (28–90 days). The relationship between the compressive strength gain and curing time followed a negative power function.
4. Within the ranges examined in this study, specimens containing appropriate amounts of superplasticizer and steel microfibers exhibited good durability under freeze–thaw cycling, indicating that the low-volume fiber reinforcement strategy does not compromise the frost resistance of SCC. However, mixtures with excessive SP dosage (1.5%) combined with high fiber volume (SCC15/4) exceeded the 5% failure threshold before 200 cycles, suggesting that not all fiber-reinforced mixtures automatically satisfy the durability requirements for structural applications.

Furthermore, two-way analysis of variance (ANOVA) confirms that steel fiber volume fraction exerts the most significant effect on both drying shrinkage and freeze–thaw resistance (*p* < 0.001), while SP dosage and their interaction also show significant effects (*p* < 0.05). This statistical evidence quantitatively demonstrates that fiber content is the dominant factor governing the durability performance of low-volume steel fiber-reinforced SCC, whereas excessive SP dosage compromises the beneficial effects of fibers through increased porosity and unfavorable pore size redistribution.

Based on a comprehensive evaluation of flowability, mechanical properties, durability, and cost, the SCC08/3 mixture (0.8% SP + 0.3 vol.% fibers) is identified as the optimal formulation for railing structure applications. This mixture satisfies all design requirements: slump flow ≥ 550 mm, 28-day compressive strength ≥ 70.1 MPa, and freeze–thaw mass loss approaching the 5% threshold at 200 cycles. Although SCC08/4 exhibits superior frost resistance, its marginal performance gain over SCC08/3 does not justify the additional fiber cost. Conversely, the 1.5% SP series consistently underperforms due to SP-induced microstructural deterioration. Therefore, SCC08/3 represents the most cost-effective and durable solution for practical railing construction.

This work presents several novel contributions. First, it systematically evaluates the combined effects of low-volume steel microfibers (0–0.4 vol.%) and two SP dosages (0.8% and 1.5%) on SCC performance within a single experimental framework—an aspect that has not been comprehensively addressed in previous investigations. Second, it provides quantitative evidence that hooked-end steel microfibers at dosages as low as 0.2 vol.% can effectively enhance flexural strength and reduce drying shrinkage, with the reduction in shrinkage being substantially greater than that reported in several earlier studies. Third, MIP analysis reveals the microstructural origins of performance deterioration induced by the higher SP dosage (1.5 wt.%), linking it to increased porosity and an unfavorable shift in pore size distribution. Taking flowability, strength, cost, and durability into consideration, the combination of 0.8% superplasticizer and 0.3 vol.% steel fibers met the requirements of C50 self-compacting concrete for railing structures, representing a cost-effective and durable solution for practical engineering applications. Fourth, two-way ANOVA provides statistical confirmation that fiber volume fraction is the most critical parameter governing both shrinkage and freeze–thaw resistance, with SP dosage and their interaction playing significant but secondary roles.

## Figures and Tables

**Table 1:** Chemical and physical properties of cement and fly ash.

	SiO_2_	Al_2_O_3_	CaO	MgO	Fe_2_O_3_	K_2_O	MnO	SO_4_	LOI	Specific Surface, Blaine, m^2^ kg^−1^	Compressive Strength, 28-Day, MPa
Cement	19.9	2.2	67.2	4.6	1.5	0.5	0.4	3.4	0.34	351	49.3
Fly ash	52.7	33.8	3.5	-	6.1	1.2	-	1.3	-	466	-

**Table 2:** Mix proportion of SCC for railing structure.

Series	Mix ID	Water-Binder Ratio	S.P. Content (%)	Steel Fibers (vol %)	Cement (kg/m^3^)	Fly Ash (kg/m^3^)	Sand (kg/m^3^)	Gravel (kg/m^3^)
Series I	SCC08/0	0.28	0.8	0	403	151	789	891
	SCC08/2			0.2	403	151	789	891
	SCC08/3			0.3	403	151	789	891
	SCC08/4			0.4	403	151	789	891
Series II	SCC15/0		1.5	0	403	151	789	891
	SCC15/2			0.2	403	151	789	891
	SCC15/3			0.3	403	151	789	891
	SCC15/4			0.4	403	151	789	891

**Table 3:** Porosity and mean radius of different mixes.

Sample	Apparent Density (g/cm^3^)	Average Pore Diameter (nm)	Total Porosity (%)	Porosity (d < 50 nm) (%)	Porosity (200 nm ≥ d ≥ 50 nm) (%)	Porosity (d ≥ 200 nm) (%)
SCC08/3	2.3557	51.09	7.75	2.45	1.54	3.76
SCC15/3	2.2809	41.58	9.14	3.29	1.92	3.93

## Data Availability

The original contributions presented in this study are included in the article. Further inquiries can be directed to the corresponding authors.

## References

[1] Thanh L., Itoh Y. (2013). Performance of curved steel bridge railings subjected to truck collisions. Eng. Struct..

[2] Yang Y., Li H., Yao G., Li L., Yang J. (2025). Study on the influence of railing ventilation rate on the aerodynamic mechanism of vortex-induced vibration in double-layer steel truss bridge. Structures.

[3] Hussain I., Ali B., Akhtar T., Jameel M.S., Raza S.S. (2020). Comparison of mechanical properties of concrete and design thickness of pavement with different types of fiber-reinforcements (steel, glass, and polypropylene). Case Stud. Constr. Mater..

[4] Frazão C., Camões A., Barros J., Gonçalves D. (2015). Durability of steel fiber reinforced self-compacting concrete. Constr. Build. Mater..

[5] Abushanab A., Vimonsatit V. (2026). Explainable machine learning and software tool for the residual compressive and tensile strength of recycled aggregate concrete at elevated temperatures. Mater. Today Sustain..

[6] Figueiredo A.D.D., Ceccato M.R. (2015). Workability analysis of steel fiber reinforced concrete using slump and Ve-Be test. Mater. Res..

[7] Abbas S., Soliman A.M., Nehdi M.L. (2015). Exploring mechanical and durability properties of ultra-high performance concrete incorporating various steel fiber lengths and dosages. Constr. Build. Mater..

[8] Afroughsabet V., Biolzi L., Monteiro P.J.M. (2018). The effect of steel and polypropylene fibers on the chloride diffusivity and drying shrinkage of high-strength concrete. Compos. Part B Eng..

[9] Li G., Zhao X., Rong C., Wang Z. (2010). Properties of polymer modified steel fiber-reinforced cement concretes. Constr. Build. Mater..

[10] Yu J., Zhang M., Li G., Meng J., Leung C.K.Y. (2020). Using nano-silica to improve mechanical and fracture properties of fiber-reinforced high-volume fly ash cement mortar. Constr. Build. Mater..

[11] Liu F., Xu K., Ding W., Qiao Y., Wang L. (2021). Microstructural characteristics and their impact on mechanical properties of steel-PVA fiber reinforced concrete. Cem. Concr. Compos..

[12] Yang X., Wang J., Zhang Z., Tan Y., Wu G. (2026). A comparative analysis of 3D-printed concrete incorporating fibers and fiber grids: Mechanical properties and microstructure. Constr. Build. Mater..

[13] Afroughsabet V., Ozbakkaloglu T. (2015). Mechanical and durability properties of high-strength concrete containing steel and polypropylene fibers. Constr. Build. Mater..

[14] Chun B., Yoo D.-Y. (2019). Hybrid effect of macro and micro steel fibers on the pullout and tensile behaviors of ultra-high-performance concrete. Compos. Part B Eng..

[15] Pang Y., Zhang Z., Wang H., Li H., Yu Q., Xu L., Chen X. (2026). Mechanical properties of hydrophobic modified steel fiber concrete under freeze-thaw cycles. Structures.

[16] Lv L.-S., Wang J.-Y., Xiao R.-C., Fang M.-S., Tan Y. (2021). Influence of steel fiber corrosion on tensile properties and cracking mechanism of ultra-high performance concrete in an electrochemical corrosion environment. Constr. Build. Mater..

[17] Mezzal S.K., Al-Azzawi Z., Najim K.B. (2021). Effect of discarded steel fibers on impact resistance, flexural toughness and fracture energy of high-strength self-compacting concrete exposed to elevated temperatures. Fire Saf. J..

[18] Roberti F., Cesari V.F., de Matos P.R., Pelisser F., Pilar R. (2021). High- and ultra-high-performance concrete produced with sulfate-resisting cement and steel microfiber: Autogenous shrinkage, fresh-state, mechanical properties and microstructure characterization. Constr. Build. Mater..

[19] Gao D., Huang Y., Yuan J., Gu Z. (2021). Probability distribution of bond efficiency of steel fiber in tensile zone of reinforced concrete beams. J. Build. Eng..

[20] Franco-Quiñonez V., Guerrero-Terán C., García-Laborda M., García-Troncoso N., Valverde-Burneo D., Galobardes I., Segura I. (2026). Mechanical properties of concrete reinforced with reused tyre steel fibres. Constr. Build. Mater..

[21] Ahmad M., Hussain Z., Akbar M., Hussain A., Lin Z., Alzara M., Yosri A.M. (2025). Self-sensing capabilities in steel fiber-reinforced concrete for shrinkage resistance and structural health monitoring. Sci. Rep..

[22] Xia Z., Geng J., Zhou Z., Liu G. (2025). Comparative analysis of polypropylene, basalt, and steel fibers in 3D printed concrete: Effects on flowability, printabiliy, rheology, and mechanical performance. Constr. Build. Mater..

[23] Alwesabi E.A.H., Bakar B.H.A., Alshaikh I.M.H., Zeyad A.M., Altheeb A., Alghamdi H. (2021). Experimental investigation on fracture characteristics of plain and rubberized concrete containing hybrid steel-polypropylene fiber. Structures.

[24] Ali B., Qureshi L.A., Shah S.H.A., Rehman S.U., Hussain I., Iqbal M. (2020). A step towards durable, ductile and sustainable concrete: Simultaneous incorporation of recycled aggregates, glass fiber and fly ash. Constr. Build. Mater..

[25] Lee S.-J., Ryu S., Won J.-P. (2021). Relationship between the rheology and flowability of self-compacting structural synthetic fibre-reinforced cementitious composites. Compos. Struct..

[26] Niş A., Eren N.A., Çevik A. (2021). Effects of nanosilica and steel fibers on the impact resistance of slag based self-compacting alkali-activated concrete. Ceram. Int..

[27] Zhao Y., Bi J., Huo L., Wang Z., Guan J., Zhao Y. (2021). Development of a coupled numerical framework of steel fiber reinforced self-compacting concrete. Constr. Build. Mater..

[28] Singh N., Singh S.P. (2016). Carbonation resistance and microstructural analysis of Low and High Volume Fly Ash Self Compacting Concrete containing Recycled Concrete Aggregates. Constr. Build. Mater..

[29] Murtaza M., Ahmad M., Mohamed M.H., Hussain W., Taqi M., Majeed A.H. (2026). Freeze–Thaw and carbonation resistance of self-compacting concrete by using partial replacement of cement kiln dust (CKD) instead of cement. Constr. Build. Mater..

[30] Golafshani E.M., Ashour A. (2016). Prediction of self-compacting concrete elastic modulus using two symbolic regression techniques. Autom. Constr..

[31] Hamad A.G., MohamadAli A.A. (2021). Effects of air post curing on recovery of bond strength and elastic modulus of fire-damaged self compacted concrete. Mater. Today Proc..

[32] Ran J., Li T., Chen D., Shang L., Li W., Zhu Q. (2021). Mechanical properties of concrete reinforced with corrugated steel fiber under uniaxial compression and tension. Structures.

[33] Teng T.L., Chu Y.A., Chang F.A., Chin H.S. (2004). Calculating the elastic moduli of steel-fiber reinforced concrete using a dedicated empirical formula. Comput. Mater. Sci..

[34] Yi P., Peng L., Liu N., Lai X., Ni J. (2013). A micromechanics elastic–plastic constitutive model for sintered stainless steel fiber felt. Mater. Des..

[35] Jin L., Zhang R., Tian Y., Dou G., Du X. (2018). Experimental investigation on static and dynamic mechanical properties of steel fiber reinforced ultra-high-strength concretes. Constr. Build. Mater..

[36] Basu P., Chandra Gupta R., Agrawal V. (2020). Effects of sandstone slurry, the dosage of superplasticizer and water/binder ratio on the fresh properties and compressive strength of self-compacting concrete. Mater. Today Proc..

[37] Benaicha M., Hafidi Alaoui A., Jalbaud O., Burtschell Y. (2019). Dosage effect of superplasticizer on self-compacting concrete: Correlation between rheology and strength. J. Mater. Res. Technol..

[38] Lalitha Surya Tejaswini G., Venkateswara Rao A. (2020). A detailed report on various behavioral aspects of self-compacting concrete. Mater. Today Proc..

[39] Vivek Sukh A., Hooda Y., Derit Singh H. (2023). Relative experimental evaluation of properties of concrete with addition of super–Plasticizers. Mater. Today Proc..

[40] (2024). Cement Part 1: Composition, Specifications and Conformity Criteria for Common Cements.

[41] Yu J., Lu C., Leung C.K.Y., Li G. (2017). Mechanical properties of green structural concrete with ultrahigh-volume fly ash. Constr. Build. Mater..

[42] (2012). Technical Specification for Application of Self-Compacting Concrete.

[43] (2019). Standard for Test Methods of Concrete Physical and Mechanical Properties.

[44] Yoo D.-Y., Banthia N., Lee J.-Y., Yoon Y.-S. (2018). Effect of fiber geometric property on rate dependent flexural behavior of ultra-high-performance cementitious composite. Cem. Concr. Compos..

[45] (2020). Test Methods of Cement and Concrete for Highway Engineering.

[46] (2025). Standard for Test Methods of Long-Term Performance and Durability of Ordinary Concrete.

[47] Yu R., Spiesz P., Brouwers H. (2014). Mix design and properties assessment of ultra-high performance fibre reinforced concrete (UHPFRC). Cem. Concr. Res..

[48] Wu Z., Shi C., Khayat K.H. (2019). Investigation of mechanical properties and shrinkage of ultra-high performance concrete: Influence of steel fiber content and shape. Compos. Part B Eng..

[49] Wu Z., Shi C., He W., Wu L. (2016). Effects of steel fiber content and shape on mechanical properties of ultra high performance concrete. Constr. Build. Mater..

[50] Yunsheng Z., Wei S., Sifeng L., Chujie J., Jianzhong L. (2008). Preparation of C200 green reactive powder concrete and its static–dynamic behaviors. Cem. Concr. Compos..

[51] Zhao M., Feng H., Wang H., Li C., Ding X., Zhao M. (2026). A study of high-flowability steel fiber reinforced small-aggregate concrete with high-resistance to early cracking and shrinkage for paving layer of slab/plates. Constr. Build. Mater..

[52] Aruntaş H.Y., Cemalgil S., Şimşek O., Durmuş G., Erdal M. (2008). Effects of super plasticizer and curing conditions on properties of concrete with and without fiber. Mater. Lett..

[53] Fan L., Meng W., Teng L., Khayat K.H. (2020). Effects of lightweight sand and steel fiber contents on the corrosion performance of steel rebar embedded in UHPC. Constr. Build. Mater..

[54] Ren G.M., Wu H., Fang Q., Liu J.Z. (2018). Effects of steel fiber content and type on static mechanical properties of UHPCC. Constr. Build. Mater..

[55] El Ouni M.H., Shah S.H.A., Ali A., Muhammad S., Mahmood M.S., Ali B., Marzouki R., Raza A. (2022). Mechanical performance, water and chloride permeability of hybrid steel-polypropylene fiber-reinforced recycled aggregate concrete. Case Stud. Constr. Mater..

[56] Li Z., Perez Lara M.A., Bolander J.E. (2006). Restraining effects of fibers during non-uniform drying of cement composites. Cem. Concr. Res..

[57] Afroughsabet V., Teng S. (2020). Experiments on drying shrinkage and creep of high performance hybrid-fiber-reinforced concrete. Cem. Concr. Compos..

[58] Barra B., Hoseinian S.B., Beygi M.A. (2003). Shrinkage of concrete stored in natural environments. Cem. Concr. Compos..

[59] Wang J., Zhao C., Li Q., Song G., Hu Y. (2025). The synergistic effect of recycled steel fibers and rubber aggregates from waste tires on the basic properties, drying shrinkage, and pore structures of cement concrete. Constr. Build. Mater..

[60] Mermerdaş K., İpek S., Algın Z., Ekmen Ş., Güneş İ. (2020). Combined effects of microsilica, steel fibre and artificial lightweight aggregate on the shrinkage and mechanical performance of high strength cementitious composite. Constr. Build. Mater..

[61] Wang L., Jin M., Wu Y., Zhou Y., Tang S. (2021). Hydration, shrinkage, pore structure and fractal dimension of silica fume modified low heat Portland cement-based materials. Constr. Build. Mater..

[62] Chakraborty D., Patey G.N. (2013). Evidence that crystal nucleation in aqueous NaCl solution Occurs by the two-step mechanism. Chem. Phys. Lett..

